# Citrate of Soda in the Feeding of Infants

**Published:** 1906-04-21

**Authors:** 


### CITRATE OF SODA IN THE FEEDING OF INFANTS.

The formation of hard curd in the stomach of an infant has long been recognised as an important factor in maintaining dyspeptic troubles. More than twelve years ago Professor E. A. Wright wrote an article suggesting the use of citrate of soda, with the view of precipitating the calcium salts in the milk, and thus preventing coagulation, the calcium salts being necessary for such coagulation. Variot [1] adopted Professor Wright's idea, and several other physicians to children's hospitals have followed his example. Dr. H. L. Keith Shaw, of New York, publishes a fresh communication on the subject. Dr. Shaw first made several experiments in order to prove whether citrate of soda really prevents the coagulation of milk, and, having obtained satisfactory results, adopted this method of treatment. Apparently he employed larger doses of citrate of soda than are often given Usually he gave as much as one grain to an ounce of milk, but sometimes as much as ten grains. His results were more encouraging than he anticipated, the control of vomiting being one of the most striking features of the treatment. There can be no doubt that citrate of soda is a useful aid in many cases of wasting in children, especially when vomiting is difficult to restrain.

[1] Archives of Pediatrics, March 1906.

